# Exertional Heatstroke Encephalopathy With Chronic Neurological Deficit

**DOI:** 10.7759/cureus.72257

**Published:** 2024-10-24

**Authors:** Manasi Harale, Sreevidya Yekkaluru, Tushar Pancholi, Arun B Oommen, Akhilesh Jagirdar

**Affiliations:** 1 General Medicine, Dr. D Y Patil Medical College, Hospital and Research Centre, Dr. D Y Patil Vidyapeeth (Deemed to be University), Pune, IND

**Keywords:** encephalopathy, focal seizures, heatstroke, hyperthermia, hypovolemic shock

## Abstract

Heat exhaustion progresses to heat stroke and then on to heatstroke encephalopathy, a serious illness. Extreme hyperthermia (over 40.5 °C), central nervous system failure, multiorgan dysfunction, and hypovolemic shock are the hallmarks of the clinical presentation of heatstroke. A 27-year-old male was presented to the causality with loss of consciousness followed by altered sensorium while working outdoors (outside temperature was 40°C), low blood pressure (70/50 mmHg), hyperthermia (41°C), tachycardia, focal seizures (1 episode), hypovolemic shock. At presentation, the Glasgow Coma Scale was E1V1M1, and he was intubated, moved to the intensive care unit, and monitored closely. He was treated with antibiotics, anticonvulsants, intravenous fluids, vasopressor supports, and body surface cooling methods. The hematological investigations showed thrombocytopenia, deranged liver, and renal function tests. On day 1, magnetic resonance imaging (MRI) of the brain showed normal study. As the patient’s neurological status showed no improvement MRI of the brain was repeated on day 8 which showed restricted diffusion with hyperintensities involving bilateral caudate nuclei, anterior aspects of bilateral putamen, and insular cortex, suggesting to rule out infective encephalopathy. Based on history, clinical presentation, laboratory, and radiological investigations this case has been diagnosed as exertional heatstroke encephalopathy.

## Introduction

When the body is unable to maintain thermoregulation, it results in heatstroke. Heatstroke is a medical condition that is defined by severe hyperthermia (body temperature between 40°C and 40.5°C), malfunctioning of the central nervous system, and failure of multiple organs [1]. There are two types of heatstroke: classic (passive) and exertional, depending on what caused it. Exertional heatstroke encephalopathy is characterized by tachycardia, tachypnea, hypotension, diaphoresis, and expanded pulse pressure. Cerebellar Purkinje cells are destroyed by direct heat injury, which also results in changes to brain perfusion, vasodilation, cerebral edema, microvascular abnormalities, and ischemic changes [2]. Diffuse cerebellar atrophy and hyperintense lesions in the dentate nuclei, bilateral superior cerebellar peduncles (SCPs), thalami, caudate nuclei, hippocampi, cerebellum, and cerebral cortices are described in diffuse weighted MRI reports [3]. Heatstroke patients should get conservative, symptomatic care. Delays in cooling can lead to severe liver disease, kidney failure, and disseminated intravascular coagulation [4]. If the patient’s neurological condition does not improve as anticipated, a high degree of clinical suspicion for heatstroke encephalopathy should be taken into consideration. Due to frequent misdiagnosis, the true prevalence of heatstroke is unknown.

## Case presentation

**Figure 1:**
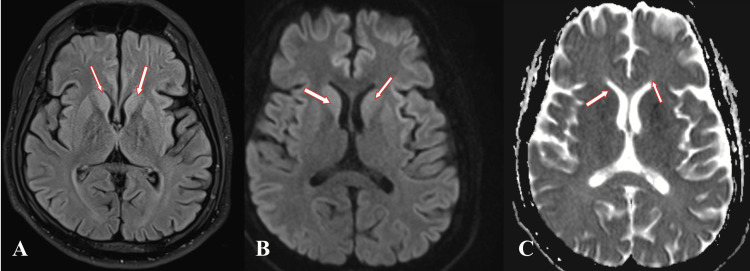
MRI brain with contrast of patient showing encephalitis changes. Hyperintense signals on FLAIR image (A) noted involving bilateral caudate nuclei, anterior aspect of bilateral putamen, and bilateral insular cortex (shown by red arrow), showing diffusion restriction on diffusion-weighted imaging sequence (B) with corresponding low apparent diffusion coefficient value (C). FLAIR: Fluid-Attenuated Inversion Recovery

A 27-year-old male was brought by relatives to causality with a sudden onset of giddiness followed by loss of consciousness for an hour and then altered sensorium. The patient’s relative gave a history of strenuous activity in the hot atmosphere in the month of April from 3 to 4 days. High-grade fever with rectal core body temperature (106°F) was recorded with hypotension, tachycardia, and one episode of focal seizures. His Glasgow Coma Scale score on presentation was E1V1M1 with quadriplegic. He was treated with intensive body surface cooling methods, intravenous fluids, vasopressor support, IV antibiotics, and anticonvulsants. Magnetic resonance imaging (MRI) brain with whole spine screening revealed no abnormality on day 1. He was evaluated with routine labs (Table 1) and arterial blood gas analysis (Table 2) revealed rhabdomyolysis, myoglobinuria, lactic acidosis with deranged liver, and renal function test. Cerebrospinal fluid analysis (CSF) studies on day 1 showed normal study. The patient’s blood pressure improved gradually and vasopressor support stopped on day 2. Furthermore, he developed fluctuating sensorium with irrelevant talks but obeyed simple commands intermittently, spontaneous eye opening, quadriparesis, and bilateral plantar were mute. He was extubated on day 3 with improvement in vitals and the Glasgow Coma Scale. In view of no as expected neurological improvement MRI brain with contrast (Figure 1) was repeated on day 8 which was described below (Figure 1). The repeat CSF study was normal and ruled out infective etiology. Nerve conduction velocity studies showed no significant changes. The electroencephalogram (EEG) did not show any epileptic form discharges. At a subsequent time, the patient showed minor neurological improvement with quadriparesis, muscle power 2/5, dysphagia, and oromandibular dyskinesia. Supportive treatment including physiotherapy was given. The patient was able to perform his daily routine after 5 months and was still receiving physical therapy at the follow-up visits.

**Table 1:** Laboratory investigations

Lab Parameters	Report	Reference Value
Hemoglobin	12 g/dL	11.6-15.0 g/dL
Total leucocyte count	8000/µL	4000-10,000/µL
Platelet count	70,000/µL	1,50,000-4,10,000/µL
Hematocrit	55%	40-54%
Random blood sugar level	82 mg/dL	70-140 mg/dL
Serum urea	90 mg/dL	17-49 mg/dL
Serum creatinine	2.5 mg/dL	0.6-1.2 mg/dL
Serum sodium	138 mmol/L	136-145 mmol/L
Serum potassium	3.9 mmol/L	3.5-5.10 mmol/L
Serum phosphorus	4 mg/dL	2.6-4.7 mg/dL
Serum magnesium	2.2 mg/dL	1.6-2.6 mg/dL
Serum bilirubin	1.5 mg/dL	0.22-1.22 mg/dL
Serum glutamic oxidative transaminase (SGOT)	100 U/L	8-43 U/L
Serum glutamic pyruvic transaminase (SGPT)	121 U/L	7-45 U/L
Total protein	7.3 g/dL	6.3-8.3 g/dL
Serum albumin	4 g/dL	3.5-5.2 g/dL
Serum globulin	3.3 g/dL	2.3-3.5 g/dL
C-reactive protein	44 mg/L	5 mg/L
Erythrocyte sedimentation rate	60 mm/h	20 mm/h
Serum procalcitonin	0.2 ng/mL	0.08 ng/mL
D-dimer	300 ng/mL	<500 ng/mL
Fibrinogen	180 mg/dL	200-400 mg/dL
Creatinine phosphokinase	200 mcg/dL	10-120 mcg /dL
Urine myoglobin	40 mg/dL	<10 mg/dL

**Table 2:** Arterial blood gas analysis Suggestive of metabolic acidosis with lactic acidosis.

Parameters	Values	Reference Value
pH (potential of hydrogen)	7.2	7.35-7.45
po_2_ (partial pressure of oxygen)	100 mmHg	75-100 mmHg
pco_2_ (partial pressure of carbon dioxide)	42 mmHg	35-45 mmHg
Lactate level	11 mmol/L	2-4 mmol/L

## Discussion

Elevation of the core body temperature above 40°C, central nervous system malfunction, and multiorgan dysfunction are the hallmarks of heatstroke the most severe heat disease in which the homeostatic thermoregulatory mechanism fails [5,6]. The anterior thalamus regulates body temperature, which is connected to the pathophysiology of exertional heatstroke [7]. In this case patient had myoglobinuria, rhabdomyolysis, and acute renal failure, and lesions in the bilateral caudate nuclei, bilateral insular cortex, and anterior parts of the bilateral putamen are visible on MRI brain with contrast [5]. Conversely, lesions in certain cases affect the frontal lobe, bilateral cerebral, cerebellar, and thalamus [3,8]. However MRI brain with contrast was ineffective in the early phase, it later showed signs of encephalopathy, and the patient’s persistent neurological symptoms were probably brought on by MRI brain abnormalities. This case showed that heatstroke can cause severe neurological deficits to the extent of quadriplegia, dyskinesia, and cranial nerve involvement in spite of normal MRI findings in the early phase. Cerebellar ataxia, dysphagia, seizures, illogical behavior, irritability, and cranial nerve abnormalities are just a few of the serious consequences that could arise as the central nervous system is highly sensitive to heatstroke [9]. Above 107°F heat can rapidly produce direct cellular injury leading to thermosensitive enzymes’ non-functional, irreversible uncoupling of oxidative phosphorylation. Delayed cooling leads to vascular endothelial damage causing coagulation abnormalities (disseminated intravascular coagulation), severe hepatic dysfunction, and multisystem organ failure where a patient requires intensive care [10,11].

## Conclusions

In order to avoid major problems, heatstroke must be diagnosed and treated quickly. Decision-making can be complicated by MRI's poor sensitivity and the ongoing discussion over when to use it to guide treatment for heatstroke encephalopathy. The neurological deficiencies noted in this case were not supported by the ambiguous MRI results. Thus, the primary approach to diagnosing and treating heatstroke encephalopathy should continue to be clinical examination. Aberrant MRI signals should be regarded as supplemental to clinical results, even though they might have academic relevance. In the end, the diagnosis and treatment plans should be based on the symptoms of the patient. A noteworthy feature of our case is the presence of typical lesions observed in the caudate nuclei, which add to its unique clinical appearance.
